# The role of the bidirectional regulatory network between immune cells and stromal cells in cardiac repair and fibrosis following myocardial infarction

**DOI:** 10.3389/fimmu.2026.1801258

**Published:** 2026-06-17

**Authors:** Fuyuan Zhang, Yiying Liu, Ruikang Liu, Baohua Li, Jun Li

**Affiliations:** 1 Guang’anmen Hospital, China Academy of Chinese Medical Sciences, Beijing, China; 2 China Academy of Chinese Medical Sciences Eye Hospital, Beijing, China

**Keywords:** cardiac fibrosis, cell communication, heart failure, immune cells, myocardial infarction, stromal cells, treatment

## Abstract

Heart failure following myocardial infarction (MI) is a major complication affecting long-term prognosis of patient, with its core pathological processes being ventricular pathological remodeling and fibrosis. This process is not merely simple scar formation, but is dominated by a continuous, dynamic, and intricate bidirectional dialogue between immune cells and cardiac stromal cells. This review systematically mapped the complex interactive networks between immune cells—including neutrophils, macrophages, and lymphocytes—and stromal cells such as cardiac fibroblasts, endothelial cells, and pericytes during the spatiotemporal evolution following MI. We emphasizes the key communication pathways involving cytokines, chemokines, fibrotic signals, and active signals from the microenvironment. And we mappied their spatiotemporal network governing the initiation and resolution of inflammation and fibrosis. Simultaneously, we explored how this network’s imbalance shifts from essential repair responses to pathological fibrosis, ultimately leading to heart failure. Furthermore, we summarized novel therapeutic strategies targeting the immune-matrix axis, aiming to provide new perspectives and theoretical foundations for the precise prevention and treatment of heart failure following MI.

## 1 Introduction

Epidemiological data indicated that nearly 9 million people die annually from ischemic heart disease, particularly myocardial infarction (MI) (1). Although the widespread adoption of reperfusion therapy has significantly reduced MI mortality, the incidence of heart failure following MI has conversely increased, substantially elevating patients’ mortality risk (2). It is estimated that approximately 20-30% of acute MI survivors develop heart failure within 1–5 years, representing a major global public health challenge (3, 4). Therefore, gaining a deeper understanding of the pathophysiological mechanisms underlying the progression from acute myocardial injury to chronic heart failure after MI, and developing effective intervention strategies, constitute urgent scientific priorities in cardiovascular research.

Traditionally, cardiac repair following MI has been described as a relatively linear, ordered cascade of events: necrosis, inflammation, proliferation, maturation, and remodeling (5, 6). While this classical linear model explains changes at different stages post-MI, it leaves a fundamental paradox: why does this process, intended to repair damage, frequently “run amok,” ultimately evolving into pathological remodeling that drives chronic heart failure? Recent advances in single-cell omics, spatial transcriptomics, and advanced imaging technologies have driven a profound paradigm shift in our understanding of the post-MI (7, 8). Cardiac repair is no longer viewed as a simple, unidirectional linear cascade but is redefined as a highly dynamic, bidirectionally interacting complex cellular network jointly governed by the immune system and the cardiac stroma (9). Within this network, immune cells and stromal cells do not engage in a simple “stimulus-response” relationship. Instead, they form a dynamically balanced ecosystem through continuous, bidirectional signaling dialogues. This ecosystem precisely regulates the initiation and resolution of inflammation, the formation of scar tissue and fibrosis, and the adaptive responses of distant myocardium across both temporal and spatial dimensions (10).

**Figure 1:**
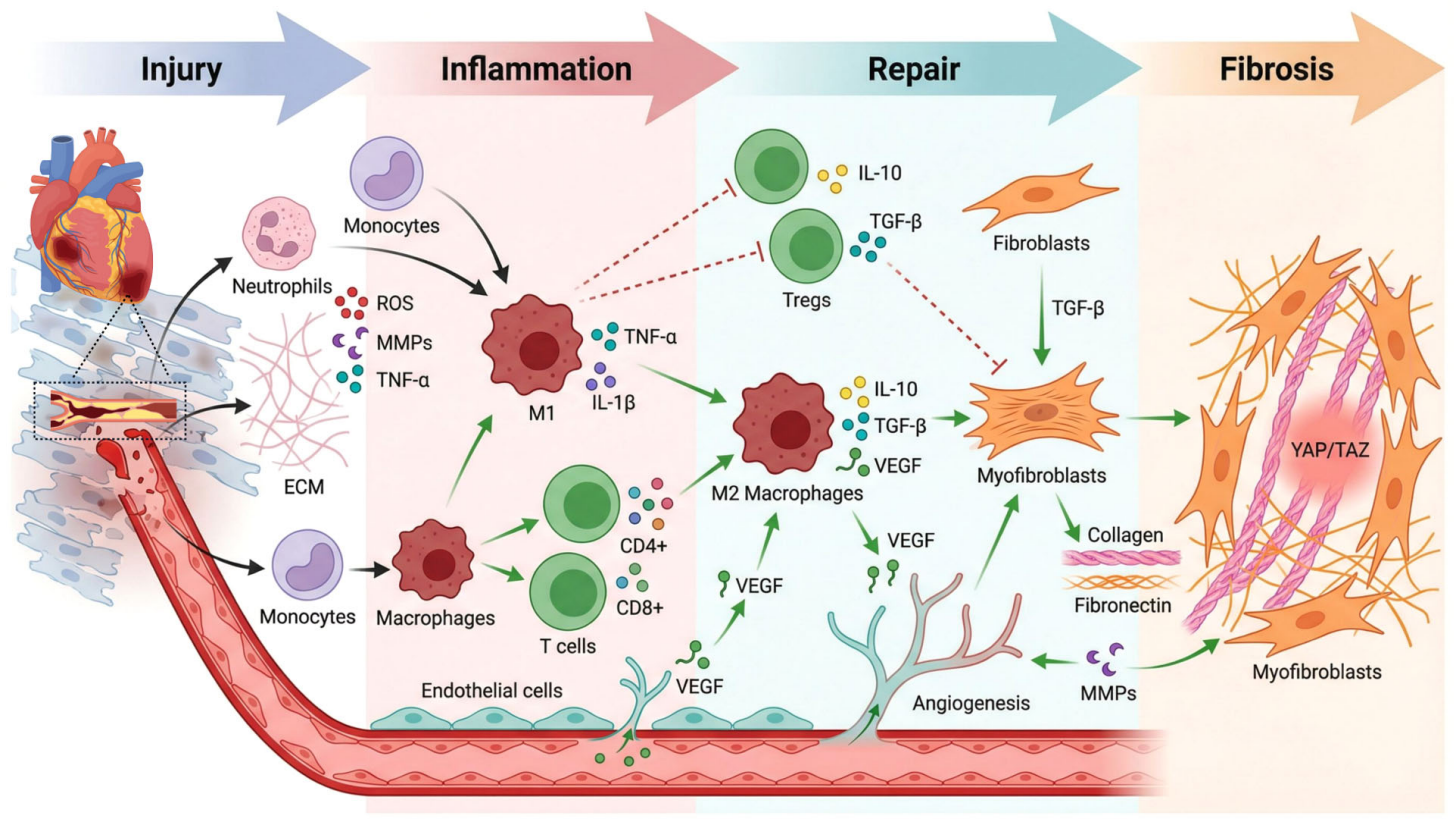
Graphical abstract. The role of the bidirectional regulatory network between immune cells and stromal cells in cardiac repair and fibrosis following myocardial infarction.

Based on this paradigm shift, this review aimed to systematically map and deconstruct the “immune-stromal cell bidirectional regulatory network” after MI. By detailing the various interacting immune and stromal cell types, elucidating key molecular mechanisms mediating intercellular communication, and mapping the network’s dynamic evolution across acute injury, transitional repair, and chronic remodeling phases, we reveal critical inflection points where orderly repair transitions to dysregulated imbalance (**Figure 1**). Furthermore, grounded in our understanding of the network’s mechanisms, we propose novel therapeutic strategies targeting this network. This review offers readers a fresh perspective on the pathogenesis and progression of heart failure following MI, laying a robust theoretical foundation for developing breakthrough therapies capable of reversing pathological remodeling and achieving cardiac repair.

## 2 Core cells in interactive networks

**Figure 2:**
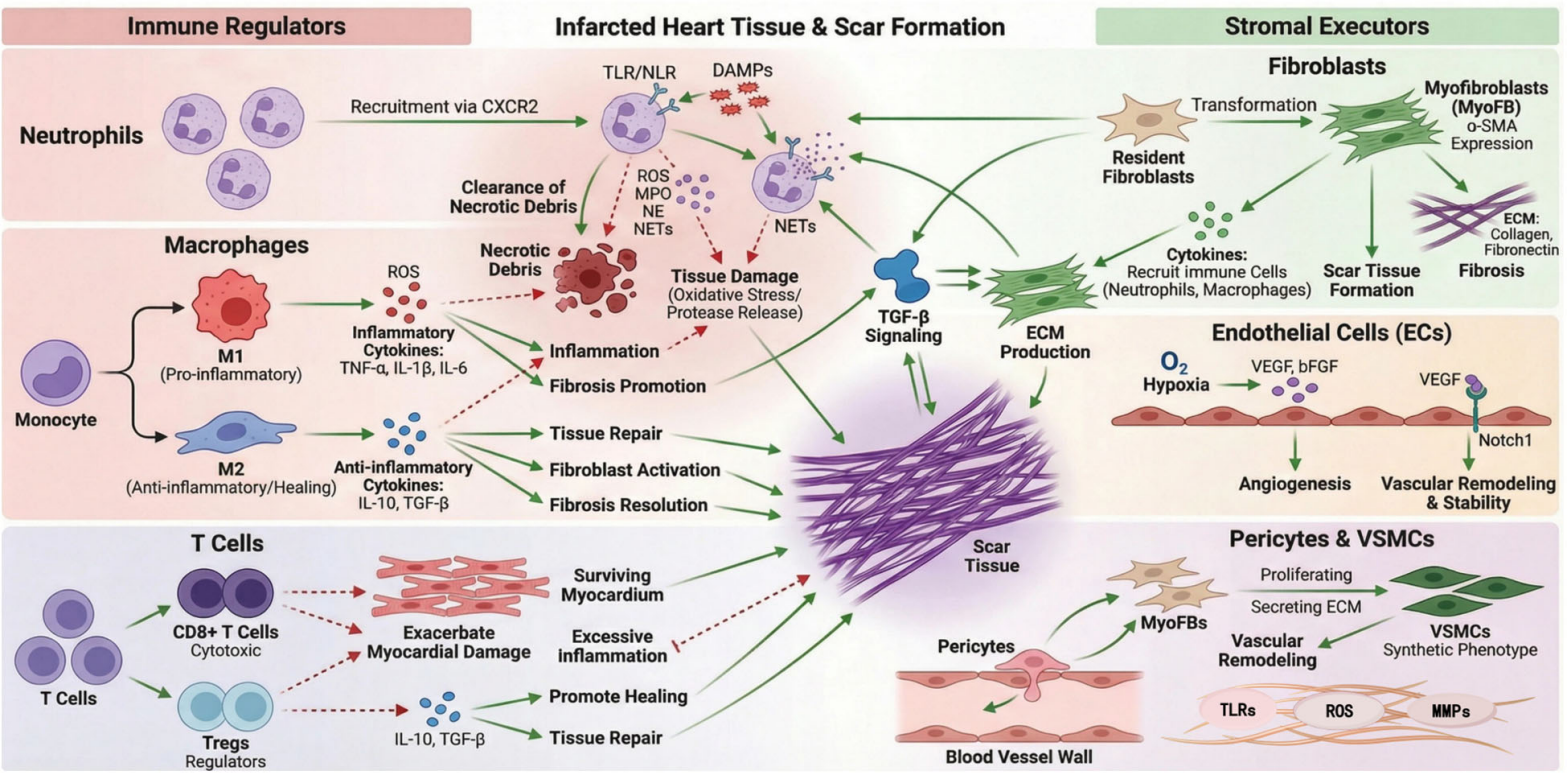
Core cells in interactive networks.

The body undergoes a series of complex and interrelated pathophysiological processes after MI. During this process, immune cells and stromal cells play distinct roles and engage in dynamic crosstalk, which collectively determine the extent of cardiac repair and fibrosis, and even influence the long-term progression of heart failure (**Figure 2**).

### 2.1 Immune cells

#### 2.1.1 Neutrophils

During the acute injury stage after MI, pattern recognition receptors (PRRs) including Toll-like receptors (TLRs) and NOD-like receptors (NLRs) recognize damage-associated molecular patterns (DAMPs) released by necrotic cardiomyocytes, thereby inducing robust production of inflammatory cytokines and chemokines (11–13). These chemokines bind to receptors such as C-X-C chemokine receptor type 2 (CXCR2) on neutrophils and drive their recruitment to the infarcted myocardium to degrade and clear necrotic cellular debris (14). Notably, neutrophils exert dual roles. Oxidants and proteases secreted by these cells not only target necrotic tissues, but also cause off-target damage to adjacent viable cardiomyocytes and the extracellular matrix (ECM) (15, 16). More importantly, while neutrophil extracellular traps (NETs) secrete harmful substances, they also act as potent DAMPs to amplify inflammatory responses and directly injure endothelial cells (ECs) (17).

The opposing functions of neutrophils in cardiac repair after MI stem from their plasticity. With the advancement of technologies such as single-cell sequencing, an increasing number of neutrophil subsets have been identified. Among them, the N1 and N2 subsets have been extensively investigated. In the acute injury stage of MI, N1 neutrophils predominate and drive macrophages to polarize toward the M1 phenotype (18). This is accompanied by elevated levels of pro-inflammatory cytokines, reactive oxygen species (ROS) and nitric oxide (NO), as well as enhanced activities of proteases and matrix-degrading enzymes (19). Starting from day 5 after MI, N2 neutrophils gradually accumulate. They promote macrophages to switch to a reparative phenotype, upregulate interleukin-10 (IL-10) and transforming growth factor-β (TGF-β), improve cardiomyocyte viability and facilitate tissue repair (18, 20). Inducing N2 polarization represents a promising strategy to accelerate inflammation resolution and tissue repair (21). After the acute phase, mesenchymal stromal cells (MSCs) promote the polarization of pro-inflammatory N1 neutrophils toward the reparative N2 phenotype via paracrine signaling, thereby modulating post-infarction cardiac repair (22). Two additional subsets, Ly6G^hi^CXCR2^+^ and Ly6G^lo^CCR2^+^, which exhibit similarities to N1 and N2 neutrophils respectively, have also been identified in the hearts of MI mice (23).

#### 2.1.2 Macrophages

Similar to neutrophils, after PRRs are activated by DAMPs, monocytes are recruited to the infarcted region under the guidance of chemokine signals (24). These cells then differentiate into primitive macrophages, which rapidly recognize apoptotic markers via surface receptors and clear necrotic cardiomyocytes and cellular debris (25). Notably, monocytes are not the sole source of cardiac macrophages. Cardiac resident macrophages derived from embryonic precursors also constitute an important population (26). Under physiological conditions, these cells reside in the heart and participate in immune surveillance and tissue homeostasis. Following acute MI, the number of resident macrophages drops sharply and they are largely replaced by monocyte-derived macrophages (27).

Macrophage polarization is a core mechanism underlying their biological functions. In the early stage of MI, macrophages are stimulated by lipopolysaccharide (LPS), interferon-γ (IFN-γ) and granulocyte-macrophage colony-stimulating factor (GM-CSF), which activates the Signal Transducer and Activator of Transcription (STAT) 1 (28), Nuclear Factor kappa-light-chain- enhancer of activated B cells (NF-kB) (29) and Hippo signaling pathways (30), which drive M1 polarization. These M1 macrophages perpetuate the inflammatory response initiated by neutrophils, further clear cellular debris, produce ROS, and secrete additional pro-inflammatory factors (31). Upon entering the proliferative repair phase, cytokines including IL-4 and IL-13 regulate M2 polarization through the PPAR-γ, STAT6 and NRF2 signaling pathways (32). They exert anti-inflammatory effects and secrete reparative mediators to promote fibroblast activation, angiogenesis and tissue repair (33).

Nevertheless, the traditional M1/M2 binary classification is insufficient to describe the complex states of macrophages. It is increasingly recognized that a macrophage population with a continuous phenotypic spectrum exists in the heart following MI. Each of these subsets exerts distinct functions at different time points post-MI (34). CCR2^+^MHC II^high^ and TIMD4^−^MHC II^low^ macrophages reside in the myocardium and are activated via TLR signaling within hours after MI, recruiting monocytes and neutrophils to the injured heart (24, 35). CCR2^high^ macrophages (36) and Dcn^hi^ macrophages (37) are primarily activated to drive rapid M1 polarization, exacerbating inflammation and myocardial injury through the release of DAMPs. The numbers of Spp1^hi^ macrophages and Ltc4s^hi^ macrophages peak on the 3rd day post-MI. MHC II^high^ macrophages and Dcn^hi^ macrophages participate in antigen processing and collagen fiber organization, with their levels peaking on the 7th day. As key players in myocardial tissue repair, TREM2^+^ macrophages gradually become predominant 7 days post-MI. They accelerate cardiomyocyte proliferation, reduce apoptosis (38) by limiting CD8^+^ T cell infiltration (39). Notably, angiotensin-converting enzyme (ACE) also plays a vital role in the immune responses of macrophages (40). Immune activation of macrophages upregulates ACE expression (41), which further modulates the metabolic function of circulating monocytes. It enhances oxidative metabolism and cellular ATP production (42), and promotes angiogenesis and matrix remodeling (43). Studies have demonstrated that ACE-associated macrophages exert vascular protective effects by upregulating peroxisome proliferator-activated receptor α and profoundly remodeling lipid metabolism (44). These findings provide novel insights into this field.

#### 2.1.3 Innate lymphoid cells and innate-like T cells

Innate lymphoid cells (ILCs) and innate-like T cells construct an early immune regulatory network after MI. They jointly secrete key inflammatory factors including IFN-γ, IL-17A and IL-4, dominate the initiation of early inflammation, and regulate the functions of macrophages, neutrophils, ECs and fibroblasts via paracrine signaling.

ILCs lack expression of T cell receptor (TCR) and B cell receptor (BCR), enabling them to directly sense microenvironmental cues and rapidly secrete cytokines. ILC1 primarily produces IFN-γ, which promotes M1 macrophage polarization and facilitates the clearance of necrotic tissues (45). ILC2 secretes IL-4, IL-5 and IL-13 to induce M2 macrophage polarization, suppress inflammation, promote angiogenesis and mediate reparative fibrosis. Meanwhile, they enhance cardiomyocyte survival by releasing growth factors (46). ILC3 secretes IL-17A and IL-22 to maintain inflammatory homeostasis and participate in tissue repair (47).

Innate-like T cells are a unique immune cell population bridging innate and adaptive immunity. Characterized by limited TCR diversity, they can rapidly respond to stress signals and serve as the major source of inflammatory factors in the early phase of acute inflammation. As the most typical subset of innate-like T cells, γδ T cells directly recognize stress molecules, lipid antigens and damage-associated molecular patterns (DAMPs). In the early stage of MI, they abundantly secrete IL-17A, IFN-γ and TNF-α, which robustly recruit neutrophils and monocytes (48). γδ T cells also exert reparative functions: they release IL-10, TGF-β and growth factors to support angiogenesis and tissue repair (49). In addition, γδ T cells cooperate with ILC2s to facilitate repair and restrain fibrosis, whereas their interaction with ILC1s amplifies inflammation and exacerbates tissue injury (50). Natural killer T (NKT) cells recognize lipid antigens and rapidly release pivotal cytokines post infarction to initiate inflammatory responses and modulate macrophage polarization (51). Mucosa-associated invariant T (MAIT) cells sense microbial metabolites and stress signals, and promptly secrete IL-17A, IFN-γ and IL-22 after MI. They participate in early inflammatory responses, regulate ECs activation and neutrophil recruitment, and contribute to the restoration of tissue homeostasis via the secretion of reparative factors (52).

#### 2.1.4 Adaptive immune cells

Adaptive immune cells, particularly T and B lymphocytes, respond relatively slowly. Nevertheless, they can participate in the early responses to MI via pre-sensitization and accumulation of senescent immune cells. Most patients with MI have a history of coronary artery disease. Long-term chronic ischemia induces stress-induced apoptosis and autophagy of cardiomyocytes, accompanied by the release of self-antigens. These antigens are captured by cardiac antigen-presenting cells, leading to pre-activation of the adaptive immune system. Guided by chemokines, the activated T cells home to and persist within cardiac tissues, becoming tissue-resident memory T cells (TRMs) (53). After acute MI, TRMs can be rapidly reactivated by local inflammatory signals or adjacent activation mechanisms without undergoing delayed processes such as lymph node priming and clonal expansion. They secrete large quantities of effector molecules within hours and aggravate early myocardial injury (54). Additionally, immune remodeling occurs with aging, accompanied by massive accumulation of senescence-associated T cells. In the early stage of MI, these cells can be directly activated by low concentrations of cytokines independent of canonical TCR signaling, thereby inducing cardiomyocyte apoptosis (55).

Following myocardial ischemia, necrosis or inflammatory injury, cardiomyocytes rupture and release large quantities of cardiac-specific autoantigens (56). These antigens are internalized and processed by resident cardiac dendritic cells (DCs), macrophages and Ly6C^hi^ monocytes, and subsequently presented on the cell surface via major histocompatibility complex class I and II (MHC-I/II) molecules (56, 57). The antigen-MHC complexes are transported through lymphatic vessels to the mediastinal draining lymph nodes, where naive CD4^+^ T cells are activated. IFN-γ secreted by Th1 cells has been shown to antagonize the profibrotic activity of TGF-β and inhibit collagen synthesis in cardiac fibroblasts (49, 58). As key adaptive immune subsets driving fibrosis, Th2 cells produce IL-4, IL-5 and IL-13 (59). They promote M2 macrophage polarization and directly stimulate the proliferation and collagen production of cardiac fibroblasts (60). Regulatory T cells (Tregs) secrete anti-inflammatory mediators such as IL-10 and TGF-β, restrain the excessive activation of CD8^+^ T cells and macrophages, and facilitate angiogenesis and moderate fibrosis (61, 62). CD8^+^ T cells are recognized to play detrimental roles after MI (63). Self-antigens released from necrotic cardiomyocytes can be cross-presented by APCs to CD8^+^ T cells. The activated CD8^+^ T cells directly attack viable but injured cardiomyocytes in the border zone, exacerbating ventricular dilatation and cardiac dysfunction (64).

B cells are traditionally regarded as antibody-producing cells, but their role in MI is far more than that. Activated B cells differentiate into plasma cells and produce a large number of antibodies. These antibodies may activate the complement system by forming immune complexes, leading to the deposition of membrane attack complex (MAC), directly damaging cardiomyocytes and microvascular endothelium, and recruiting more inflammatory cells (65, 66). Meanwhile, as efficient professional APCs, B cells can uptake and process cardiac injury-related antigens, and present them to CD4^+^T cells through MHC II molecules, thereby initiating and amplifying antigen-specific T cell responses (67, 68). Similar to Tregs, there is a B cell subset with immunosuppressive function—regulatory B cells (Bregs). They mainly secrete inhibitory cytokines such as IL-10, IL-35 and TGF-β to inhibit the activity of inflammatory T cells and macrophages, and promote the generation of Tregs (69, 70). Transplantation of Bregs after MI reduces the infiltration of Ly6C^hi^ monocytes, thereby reducing myocardial injury (70).

### 2.2 Structural and functional pillars of cardiac stroma

The cardiac stroma constitutes the physical and biochemical microenvironment of the heart. It is composed of various ECM components and stromal cells embedded in them, jointly maintaining the structural integrity and signal transduction of the heart. After MI, the stromal system is deeply activated and remodeled by the immune response, transforming from a structural pillar maintaining homeostasis to an active signal driving repair and remodeling (71).

#### 2.2.1 Cardiac fibroblasts

CFs are the most abundant non-cardiomyocytes in the heart (72). In the quiescent state, they proliferate slowly, mainly synthesize low levels of ECM to maintain tissue homeostasis, and sense changes in the microenvironment (73). After MI, stimulated by signals such as TGF-β, platelet-derived growth factor (PDGF), receptor for advanced glycation end products (RAGE) and IL-1, CFs undergo significant phenotypic transformation into myofibroblasts (74, 75). Myofibroblasts are characterized by the expression of α-smooth muscle actin (α-SMA), formation of stress fibers, and acquisition of strong contraction, migration, proliferation and ECM synthesis capabilities (76, 77). They secrete a large amount of collagen I, collagen III and fibronectin, and are the main force for scar tissue formation (78, 79). Activated CFs are not only producers of ECM but also active immune regulatory cells. In the early stage of injury, CFs can rapidly secrete a series of chemokines such as C-X-C motif chemokine ligand (CXCL) 1, CXCL2, CXCL5 and CXCL6, recruiting a large number of neutrophils to the inflammatory site (80). They also secrete abundant pro-fibrotic and pro-inflammatory cytokines, forming a positive feedback loop to sustain fibrosis and inflammation (81, 82). They maintain the expression of M-CSF during the inflammatory and fibrotic phases. The persistent presence of M-CSF provides a key signal for macrophages to stay in fibrotic lesions, and regulates the proliferation, differentiation and migration of target immune cells through the phosphoinositide 3-kinase (PI3K)-protein kinase B (AKT), janus kinase (JAK)-STAT and mitogen-activated protein kinase (MAPK) pathways (83, 84). In addition, they regulate vascular responses, which may impair endothelial barrier function, thereby exacerbating inflammation-driven fibrosis (51). Of course, CFs in the adult heart exhibit significant functional heterogeneity. For example, some highly express platelet-derived growth factor receptor (PDGFR) α and participate in intercellular communication and paracrine signaling (85, 86). Others tend to express PDGFRβ, are more sensitive to pro-fibrotic signals, and more likely to transform into myofibroblasts (87).

#### 2.2.2 Endothelial cells

Cardiac microvascular ECs form a selective barrier between blood and myocardial tissue, and act as a dynamic sensing and signal transduction center.

The hypoxic environment after MI not only leads to cell death but also activates hypoxia-inducible factor 1α (HIF-1α). It drives ECs to secrete VEGF and basic fibroblast growth factor (FGF), promoting neovascularization, which is crucial for tissue reperfusion during the repair phase (88). VEGF can significantly attenuate left ventricular remodeling, reduce infarct size, and promote cardiomyocyte survival and angiogenesis (89). The interaction between VEGF signaling and Notch1 regulates vascular branching and endothelial lumen formation, thereby improving the density and stability of the vascular network (90, 91). Moreover, in vascular ECs, Notch regulates the expression of adhesion molecules such as vascular cell adhesion molecule-1 (VCAM-1) and intercellular adhesion molecule-1 (ICAM-1), induces leukocyte recruitment and transendothelial migration, and exerts barrier function (92, 93). ECs also secrete nitric oxide (NO), endothelial cell-specific molecule-1 (ESM-1), angiopoietin-1 (Ang-1) and prostaglandins, regulating vascular tone, platelet aggregation and local inflammation (94, 95). More importantly, in addition to regulating platelet aggregation and inflammation levels, ECs also transport essential substances such as glucose, amino acids and L-arginine to maintain homeostasis (96, 97). With the in-depth study of single-cell sequencing and other technologies, more and more ECs subtypes have been discovered (98), and the apoptosis and autophagy of ECs may also be the focus of next-step research (99).

#### 2.2.3 Other stromal cells

In addition to fibroblasts and ECs, pericytes wrapping the outer wall of microvessels and VSMCs in the wall of larger blood vessels are also involved in regulating vascular stability and endothelial function (100). Pericytes are one of the earliest “awakened” cells after MI, expressing neuron-glia antigen 2 (NG2), PDGFRα (101) and angiotensin-converting enzyme 2 (ACE2) (102). They detach from the vascular wall, proliferate, migrate, and differentiate into myofibroblasts in large quantities, being one of the main sources of myofibroblasts in infarct scars (103). Studies have shown that 16.6% of myofibroblasts after MI are derived from pericytes (85). In addition, due to the close physiological structural relationship between pericytes and ECs (shared basement membrane and gap junctions), there is direct or indirect communication between them (104). Pericytes regulate vascular stability and endothelial function by secreting factors (105). In the absence of blood flow, they drive endothelin-1 (ET-1) to affect ECs dynamics, reduce capillary extracellular matrix and detach from the microvascular wall, promoting capillary wall remodeling (106). Of course, pericytes also express TLR receptors and participate in the post-MI inflammatory environment by secreting cytokines and chemokines (107, 108). Under pathological stimulation, VSMCs switch from the “contractile phenotype” to the “synthetic phenotype”, proliferate and secrete ECM, participating in vascular remodeling and fibrosis. VSMCs can also become foam cells, showing decreased expression of α-SMA accompanied by increased macrophage markers such as CD68 (109). Studies have also shown that under hypoxic conditions, VSMCs are activated by HIF, increasing ROS production and promoting the calcification of valvular interstitial cells (VICs) (110, 111). Furthermore, VSMCs can produce and secrete MMPs, thereby enhancing the proteolytic activity against elastin and collagen, increasing the risk of plaque rupture and thrombosis, and affecting post-MI repair (112).

## 3 Molecular mechanisms mediating bidirectional communication

**Figure 3:**
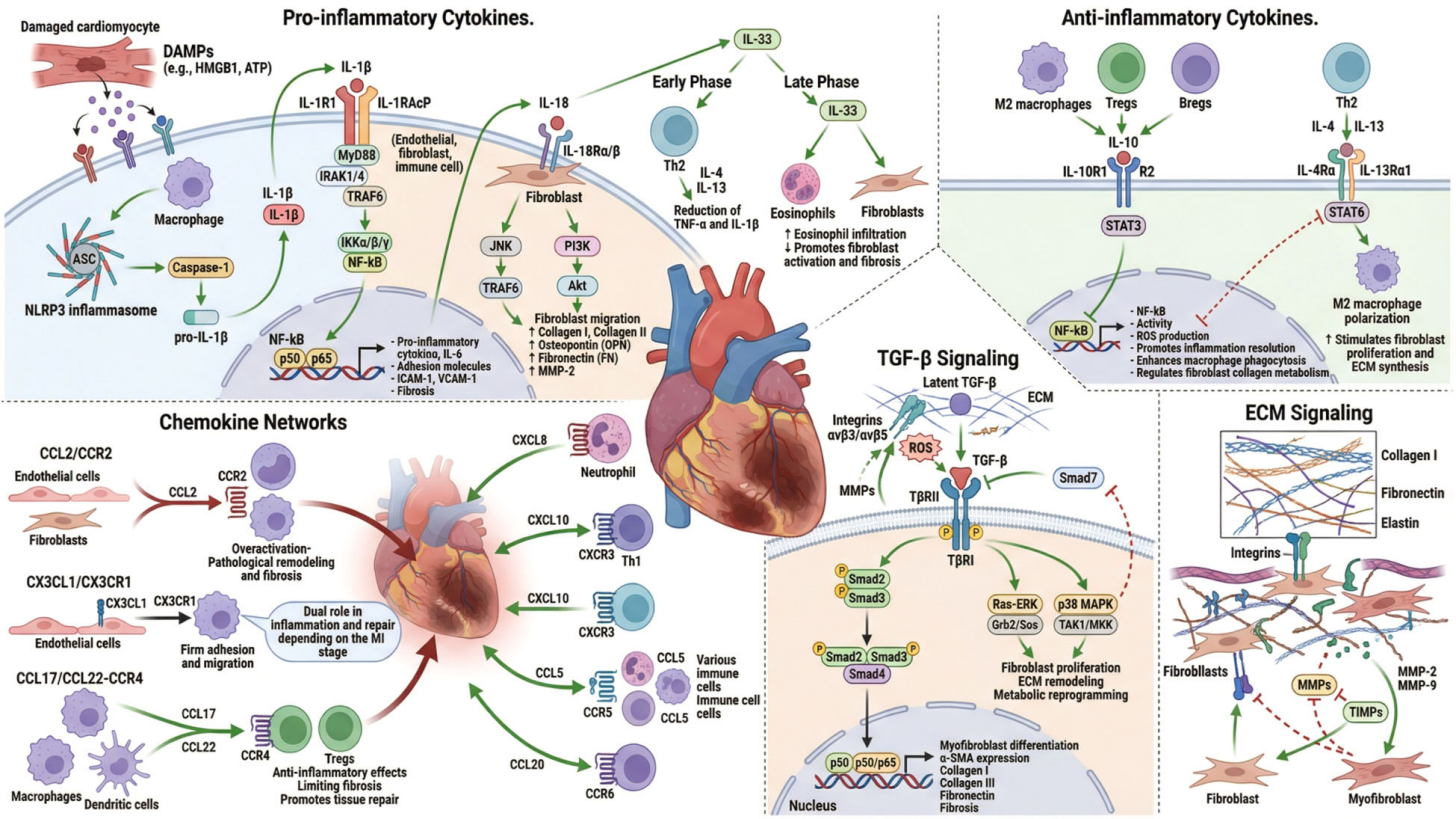
Molecular mechanisms mediating bidirectional communication.

After MI, numerous immune cells and stromal cells do not function independently. They jointly construct a complex communication network for cardiac repair and fibrosis through inflammatory cytokines, chemokines, TGF-β and active signals of the matrix microenvironment itself (**Figure 3**).

### 3.1 Inflammatory cytokines

#### 3.1.1 Pro-inflammatory cytokines

IL-1β, IL-18 and TNF-α are predominantly secreted by neutrophils, monocytes and M1 macrophages, serving as key mediators of the early inflammatory response (113, 114). After MI, DAMPs trigger excessive ROS production, which facilitates NLRP3 inflammasome assembly and caspase-1 activation. Activated caspase-1 cleaves pro-IL-1β into mature and biologically active IL-1β (115, 116). IL-1β further amplifies the inflammatory cascade and recruits neutrophils and monocytes by activating the MyD88-NF-κB signaling pathway via IL-1R1 (117). IL-18 upregulates the expression of TNF-α, IL-17 and IFN-γ in the heart by inducing CXCL16 in endothelial cells (118). TNF-α promotes inflammation, cardiomyocyte apoptosis and matrix degradation mainly through the NF-κB and MAPK pathways (119). Notably, IL-1β and IL-18 also exert impacts on cardiac repair at the late stage of MI. IL-1β acts on cardiac fibroblasts to shift them toward a matrix-degrading phenotype, upregulate fibroblast growth factors and drive collagen synthesis (74, 120). IL-18 stimulates the production of type I collagen, type II collagen, osteopontin (OPN), fibronectin (FN) and MMP-2. It activates the JNK and PI3K signaling pathways to induce fibroblast migration, proliferation and collagen deposition, thereby exacerbating fibrosis (121, 122). IL-33 plays dual roles following MI. In the early phase, IL-33 potently induces Th2 cytokine production, reduces the levels of pro-inflammatory TNF-α and IL-1β, and alleviates acute cardiac inflammation (123). However, at the chronic stage starting from day 7 post infarction, IL-33 binds to its receptor ST2L, markedly increases myocardial eosinophil infiltration, expands the infarct size, and triggers fibrosis and immune responses (124). Degradation of IL-6 mRNA suppresses pro-inflammatory effects in the heart under pressure overload (125). After MI, IL-6 activates the JAK-STAT3 pathway through its membrane receptor IL-6R (126). It sustains inflammation and upregulates TGF-β receptors on cardiac fibroblasts, which sensitizes fibroblasts and forms a vicious cycle linking inflammation and fibrosis (127). IL-17 is mainly secreted by γδ T cells, Th17 cells and ILC3s. It induces cardiomyocyte apoptosis via the STAT3-iNOS and p38 MAPK-p53-Bax signaling axes, and regulates ventricular remodeling in both the early and late phases of MI (128, 129).

#### 3.1.2 Anti-inflammatory cytokines

IL-10 is mainly produced by M2 macrophages, Tregs and Bregs. It inhibits the expression of NF-κB (130), human antigen R (HuR) and MMP-9 (131) via phosphorylation and activation of STAT3, thereby reducing the production of pro-inflammatory cytokines and ROS, and improving left ventricular function and remodeling. Meanwhile, IL-10 enhances macrophage efferocytosis, indirectly regulates collagen metabolism in fibroblasts and restricts excessive fibrosis (132). IL-4 and IL-13 serve as a critical link between Th2 immune responses and fibrosis. Predominantly secreted by Th2 cells, ILC2s and mast cells, they activate the STAT6 signaling pathway to collectively drive macrophage polarization toward the reparative M2 phenotype (133, 134). These cytokines also act directly on cardiac fibroblasts to stimulate cell proliferation and ECM synthesis (135). With the deepening of research, two new members of the IL-1 family, IL-37 and IL-38, have been discovered. They inhibit the NOTCH1 and NF-κB signaling pathways, promote the transformation of macrophages from M1 to M2, reduce collagen deposition, and increase the secretion of anti-inflammatory cytokines, thereby alleviating myocardial ischemia-reperfusion injury and myocardial fibrosis (136–140). They also synergistically enhance the activity of phosphatase and tensin homolog (PTEN), inhibit Akt, MAPK and spleen tyrosine kinase (SYK) signaling pathways, reduce ROS generation, regulate platelet activation, and prevent myocardial injury (141, 142).

### 3.2 Chemokines

Chemokines are a large family of small-molecular cytokines. By binding to their corresponding G protein-coupled receptors (GPCRs), they provide precise navigation signals for immune cells, guiding their directional migration, homing and localization (143). After MI, a sophisticated network composed of various chemokines and their receptors is rapidly activated to ensure that various immune cells are recruited to the correct location at the right time (144).

The binding of CCL2 to its primary receptor CCR2 is well recognized as the core pathway for inflammatory monocyte recruitment and an essential step in initiating the repair process (145). In the early stage of MI, damaged cardiomyocytes, activated resident macrophages, as well as endothelial cells and fibroblasts rapidly stimulated by DAMPs, are the major sources of early CCL2 production (146). Its expression is tightly regulated by key transcription factors including NF-κB, NLRP3, activator protein 1 (AP-1) and granulocyte-macrophage colony-stimulating factor (GM-CSF) (147–149). Nevertheless, uncontrolled early inflammation leads to continuous amplification of the above pro-inflammatory signals and triggers a cascade reaction, resulting in aberrantly elevated CCL2 expression and subsequent excessive activation (150). Sustained overexpression of CCL2 causes massive and excessive monocyte infiltration, leading to persistent local inflammation. Studies have verified that persistently high CCL2 levels serve as an independent risk factor for predicting left ventricular dysfunction and adverse clinical outcomes (151). Inhibiting the excessive activation of CCL2 or CCR2 can markedly reduce monocyte accumulation in the infarcted area in repair phase, alleviate left ventricular dilatation and fibrosis, and improve cardiac function (152).

C-X3-C motif chemokine ligand 1 (CX3CL1) is a chemokine with a unique structure, widely expressed in activated ECs in a membrane-bound form, and can also be produced by smooth muscle cells and certain dendritic cells. CX3CL1 can directly and strongly adhere to cells expressing its only receptor CX3C motif chemokine receptor 1 (CX3CR1), such as monocytes, T cells and NK cells, mediating the capture and firm adhesion of leukocytes (146). In the early stage of MI, membrane-bound CX3CL1 is cleaved by metalloproteinases to release a soluble form. Soluble CX3CL1 exerts classical chemotactic activity, promoting the adhesion, infiltration and migration of monocytes and CD8^+^T cells, exerting a pro-inflammatory effect in the early inflammatory response, which may exacerbate tissue damage (153, 154). Surprisingly, many studies suggest that this axis has an anti-fibrotic effect. This is mainly because CX3CR1 is an important marker of cardiac resident macrophages, which have anti-inflammatory and repair functions (155). CX3CL1 signaling helps maintain the survival and localization of these protective macrophages.

In the repair phase after MI, the focus of the immune response shifts from clearing necrotic debris to inhibiting excessive inflammation and promoting tissue remodeling. CCL17 and CCL22 play a key coordinating role in this phase by binding to CCR4. CCL17 and CCL22 are mainly produced by M2 macrophages, dendritic cells and ECs, and their core function is to recruit regulatory Tregs (156). CCL17 and CCL22 produced locally in the infarct area can efficiently recruit circulating Tregs to the injury site. The recruited Tregs exert powerful immunosuppressive and repair-promoting effects through multiple mechanisms. On the one hand, Tregs secrete anti-inflammatory factors such as IL-10 and TGF-β, directly inhibiting the activation of effector T cells and macrophages and the production of inflammatory factors (157). On the other hand, Tregs help control the degree of fibrosis and prevent excessive scar formation by regulating macrophage polarization or directly acting on fibroblasts (158). Animal studies have shown that enhancing Treg recruitment mediated by the CCL22/CCL17-CCR4 axis can improve cardiac function and reduce adverse remodeling after MI. Conversely, insufficient function of this axis may lead to immunosuppressive imbalance and persistent inflammation, which is not conducive to repair (159).

In addition to the above three core axes, the post-MI chemokine network also involves other important members, which together constitute a sophisticated regulatory system. The CXCL8-IL-8-CXCR1/2 axis is one of the initiating links of acute inflammation, mainly chemotactic for neutrophils (160). The CXCL10-CXCR3 axis is associated with pro-inflammatory and cytotoxic responses, mainly chemotactic for Th1 cells and NK cells (161, 162). The CCL5-CCR5 axis chemotaxes various immune cells such as monocytes and T cells, playing a more significant role in the chronic inflammation and fibrosis stages (163, 164). CCL20-CCR6 mainly chemotaxes the accumulation of neutrophils and macrophages, participating in the inflammatory process (165). In addition to the well-known stem cell homing function, the CXCL12-CXCR4 axis is also involved in angiogenesis, inflammation, and the regulation of cardiomyocyte survival (166).

### 3.3 Transforming growth factor-β

One consequence of pro-inflammatory is tissue repair. However, due to limited regenerative capacity, the myocardium has been shown to respond through scar formation (51). During this process, multiple immune cells are recruited to the injured site and contribute to the development of myocardial fibrosis (167). TGF-β is widely recognized as the most core regulatory factor in the process, stored in the ECM in the form of a latent complex (168). After MI, TGF-β is activated and released under the action of integrins αvβ3/αvβ5, ROS and MMP (169, 170). On the one hand, activated TGF-β recruits and binds to TGF-β receptor type I (TβRI) and TβRII on the cell surface, thereby phosphorylating downstream Smad2/3 to translocate into the nucleus, initiating the transcription of fibrosis-related genes (171), and driving the transformation of cardiac fibroblasts into myofibroblasts (172). On the other hand, TGF-β can directly phosphorylate ShcA through its kinase activity, thereby activating the rat sarcoma (Ras)-extracellular regulated protein kinases (ERK) pathway and promoting fibroblast proliferation (173). In addition, TGF-β can rapidly activate the MAPK pathway (174) and PI3K/Akt pathway (175), participating in the energy metabolism reprogramming and survival of fibroblasts. These non-Smad pathways and Smad pathways constitute a dense signaling network, jointly determining the final phenotype of cells. It is worth noting that to prevent excessive activation of TGF-β signaling, the body has a sophisticated negative feedback mechanism. Smad7 is a key molecule among them. It is induced to express by Smad signaling, and then recruits E3 ubiquitin ligase to promote the degradation of TβRI, forming a negative feedback loop (176). In addition to fibroblasts, TGF-β also interacts with various cells in the body such as monocytes/macrophages, neutrophils, ECs and cardiomyocytes. As a key regulator of immune cell phenotype and function, TGF-β may regulate cardiomyocyte survival, promote the chemotaxis of monocytes and neutrophils, and regulate the differentiation and activation of lymphocytes (177). Pro-inflammatory Ly6C^hi^ macrophages stimulate myofibroblasts through TGF-β and osteopontin, further accelerating the fibrotic response, while M2 macrophages can partially counteract this effect by producing IL-10 (178, 179). Studies have also shown that TGF-β promotes vascular maturation by regulating the interaction between ECs and pericytes, participating in the regulation of infarct angiogenesis (180).

### 3.4 Active signals of the matrix microenvironment

The ECM is not a passive structural scaffold but a dynamic and active signal library, actively participating in and guiding cellular growth and migration. Structural proteins, proteoglycans, elastin and fibronectin together form a complex network of ECM. collagen I and collagen III are not only structural proteins but also can transmit signals to cells through discoidin domain receptors (DDR) 1/2, regulating cell proliferation, differentiation and the expression of MMPs (181, 182). ECM fragments degraded by MMPs can act as endogenous DAMPs, activating innate immune cells and fibroblasts through receptors such as TLR2/4 and CD44, producing more pro-inflammatory factors and MMPs, and continuously driving inflammation and remodeling (183). Elastin is mainly involved in the regulation of tissue elasticity. Its degradation product, elastin-derived peptides (EDPs), chemotaxes monocytes and stimulates smooth muscle cell proliferation by binding to elastin receptors, participating in angiogenesis (184–186). FN mediates cell adhesion and signal transduction. Its splicing variant EDA-FN is specifically upregulated after tissue injury (187). It directly activates innate immunity through TLR4 and enhances the responsiveness of fibroblasts to TGF-β, being a key mediator of the injury response (188, 189).

After MI, excessive deposition and cross-linking of ECM lead to a significant increase in tissue stiffness. Cells sense this mechanical change through integrins and initiate intracellular mechanical signal transduction (190). Integrin clustering recruits focal adhesion proteins, activates focal adhesion kinase (FAK) and Src family kinases, forming a FAK-Src complex as the core amplifier of mechanical signal transduction, which in turn triggers a series of downstream signals (191). Yes-associated protein (YAP)/Transcriptional coactivator with PDZ-binding motif (TAZ), the core transcriptional co-activators of mechanical signals, are key hubs among them (192). On soft matrices, YAP/TAZ are in a phosphorylated state and retained in the cytoplasm. On stiff matrices, cell tension and FAK signaling inhibit large tumor suppressor 1/2 (LATS1/2) kinases, leading to dephosphorylation of YAP/TAZ and translocation into the nucleus (190, 193). In the nucleus, YAP/TAZ do not directly bind to DNA, but combine with transcription factors to initiate the expression of pro-proliferative and pro-fibrotic genes, thereby maintaining the activated state of myofibroblasts (194). This forms a vicious cycle: myofibroblasts secrete ECM to increase stiffness, and the increased stiffness in turn maintains the myofibroblast phenotype through integrin-FAK-YAP/TAZ signaling feedback.

## 4 Spatiotemporal dynamic evolution of the interaction network

**Figure 4:**
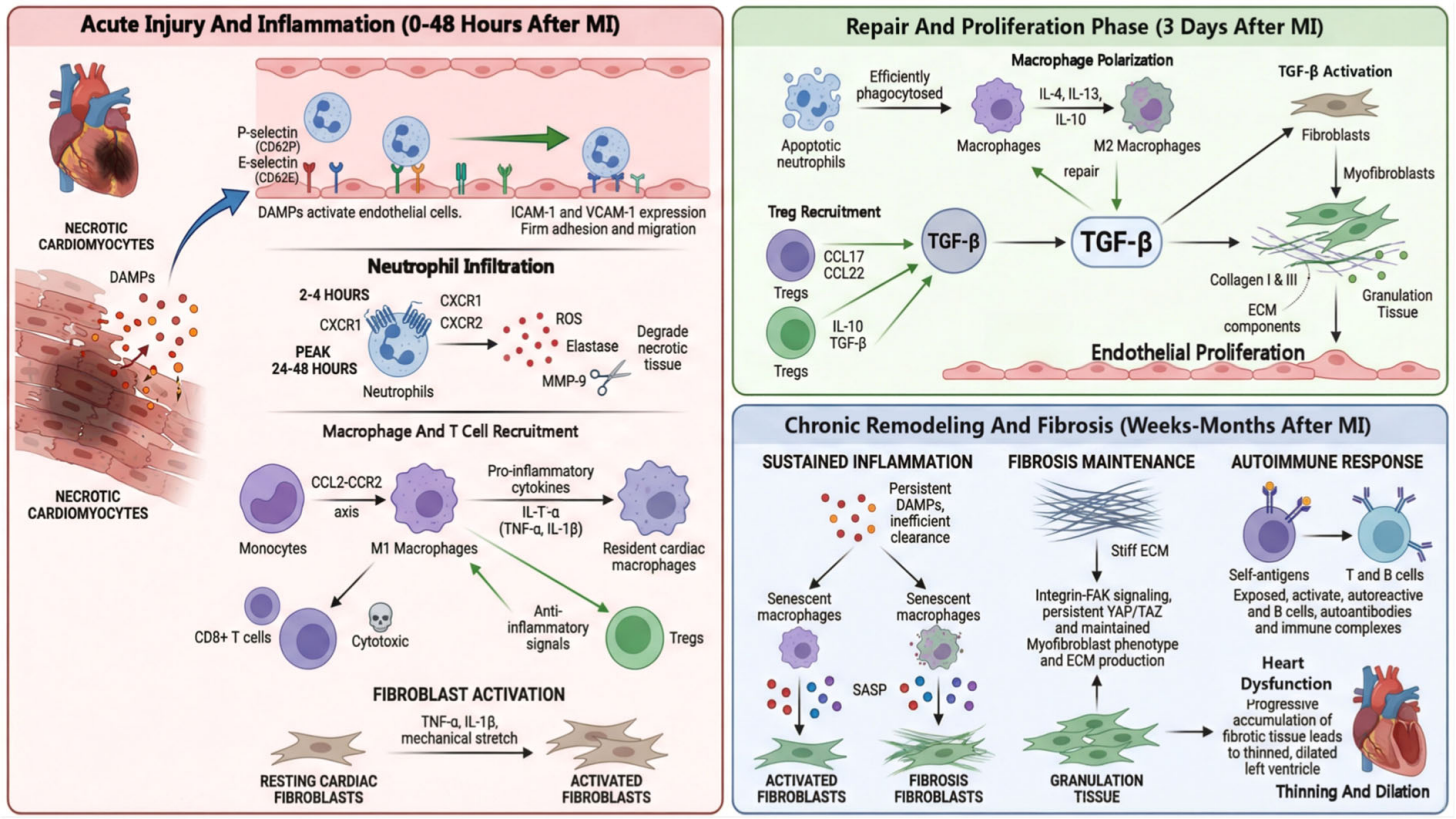
Spatiotemporal dynamic evolution of the interaction network.

Cardiac repair and remodeling after MI is not achieved overnight but a spatiotemporal interaction network bidirectionally regulated by immune-stromal cells. Its composition, activity and dominant signals dynamically evolve with disease progression. Understanding this spatiotemporal dynamic evolution is the key to revealing whether the heart ultimately moves towards stable healing or progressive failure (**Figure 4**).

### 4.1 Acute injury and inflammatory phase

The DAMPs, released by necrotic cardiomyocytes after MI, are recognized by PPRs, activating local resident cells. Stimulated by DAMPs and hypoxia, ECs respond rapidly, upregulating P-selectin and E-selectin to promote leukocyte rolling, and then inducing the expression of intercellular adhesion molecule-1 (ICAM-1) and vascular cell adhesion molecule-1 (VCAM-1) (7, 195). At the same time, endothelial barrier function is impaired and vascular permeability is increased.

Neutrophils begin to infiltrate in large quantities within 2–4 hours after MI, peaking at 24–48 hours (18). They sense chemokines released by ECs and resident cells through CXCR1 and CXCR2, release ROS, elastase and MMP9 to dissolve and clear necrotic cell debris (16, 196). Almost simultaneously, circulating inflammatory monocytes are recruited to the heart driven by the powerful CCL2-CCR2 axis. They differentiate into macrophages and become the dominant immune cells in the inflammatory phase. They secrete a large number of pro-inflammatory factors (197), and further strengthen the phagocytic clearance of necrotic debris (198). DAMPs and inflammatory cytokines also indirectly activate TRMs and senescence-associated T cells, which participate in the early regulation of MI independent of canonical TCR signaling. Both CD4^+^T and CD8^+^T cells undergo non-specific bystander activation. In addition, inflammatory signals rapidly reactivate pre-sensitized T cells. Accordingly, early T cell activation exhibits a mixed pattern predominantly driven by inflammatory factors and secondarily mediated by self-antigen specificity (54, 55). Stimulated by the pro-inflammatory microenvironment, CD8^+^T lymphocytes acquire enhanced cytotoxicity, which may cause further damage to the infarcted myocardium (199). Pro-inflammatory cytokines such as interferon-γ secreted by CD4^+^ T lymphocytes further potentiate the cytotoxic effects of CD8^+^ T cells and aggravate cardiac injury (200, 201). Of course, lymphocytes also play a positive role. Tregs secrete anti-inflammatory cytokines and mediators to alleviate ischemia-reperfusion injury (202). Quiescent cardiac fibroblasts are activated by early inflammatory factors such as TNF-α, IL-1β and mechanical stretch, start to proliferate, and express early activation markers, preparing for subsequent transformation.

### 4.2 Repair and proliferative phase

Approximately from the 3rd day after MI, the acute inflammatory state is controlled, and the heart enters the repair and proliferative phase. Neutrophils are efficiently phagocytosed and cleared by macrophages through apoptosis, and this efferocytosis is the signal switch for inflammation resolution (203). Macrophages themselves undergo profound phenotypic reprogramming, polarizing from the pro-inflammatory M1 phenotype to the reparative M2 phenotype (204). This polarization is driven by multiple signals such as apoptotic cells, IL-4, IL-13 and IL-10 (132). Meanwhile, Large numbers of Tregs are recruited to the injury site by chemokines, and inhibit excessive effector T cell responses by secreting IL-10 and TGF-β, assisting the transformation of macrophages to the repair phenotype (205, 206). ILC2s are also activated and secrete IL-13 to jointly promote a reparative environment (134, 207). TGF-β is the core promoter of the fibrosis program, and its level reaches peak during this stage. Driven by TGF-β, activated cardiac fibroblasts differentiate into myofibroblasts in large quantities. They secrete a large amount of ECM dominated by collagen I and collagen III, and together with new blood vessels, construct cell-rich granulation tissue to replace the initial necrotic area (208). In addition, under the regulation of HIF-1α, ECs proliferate and migrate to form a new capillary network, providing oxygen and nutrients for the highly metabolically active granulation tissue.

### 4.3 Chronic remodeling and fibrotic phase

For numerous patients, proliferation and tissue repair do not represent the final outcome; instead, the condition progresses into a chronic and progressive pathological state. Under the long-term influence of the microenvironment shaped by chronic inflammation and tissue injury, macrophages acquire distinct phenotypes and functions and differentiate into pro-fibrotic macrophages. These cells mainly secrete mediators such as transforming growth factor-β, which drive the activation, proliferation and collagen synthesis of fibroblasts, and sustain the fibrotic microenvironment via paracrine and mechanical signaling (209). Impaired efferocytosis and accumulation of senescent immune and stromal cells lead to continuous release of DAMPs, keeping the body in a state of low-grade but persistent inflammation for a long time (196). Incompletely cleared necrotic debris and exposed self-antigens may trigger the activation and expansion of autoreactive T and B lymphocytes. These cells respond to cardiac antigens and serve as a long-term, specific driver of inflammation. They may also produce autoantibodies to form immune complexes, which further exacerbate tissue damage (210–212). The rigid collagen matrix that has already been deposited continuously activates mechanosensitive transcription factors including YAP/TAZ through the integrin-focal adhesion kinase signaling pathway. This not only maintains the activated phenotype of myofibroblasts and sustains their ECM secretion, but also further promotes the differentiation of macrophages toward a pro-fibrotic phenotype (213). Senescent macrophages, ECs and fibroblasts also secrete a large number of inflammatory cytokines, chemokines and proteases, namely the senescence-associated secretory phenotype (SASP). SASP creates a powerful pro-inflammatory and pro-fibrotic microenvironment, being a key bridge connecting senescence, chronic inflammation and fibrosis (214). Under the combined action of the above imbalance mechanisms, the cardiac structure undergoes progressive deterioration. The scar tissue in the infarct area cannot mature and contract normally, but instead becomes thin and dilated due to continuous protease activity and mechanical stress. Remote myocardium undergoes diffuse interstitial and perivascular fibrosis under the influence of neurohumoral factors and pathological signals from the infarct area.

## 5 From network regulation to precision medicine

The imbalance of the immune-stromal cell interaction after MI is the core mechanism driving the heart towards pathological remodeling and failure. Traditional anti-inflammatory or anti-fibrotic therapies often fail due to lack of cell specificity and spatiotemporal precision, and even interfere with the necessary repair process. Therefore, precise intervention targeting this network represents a new paradigm for the treatment of heart failure following MI (**Table 1**).

**Table 1:** Therapeutic strategies for modulating immune responses in MI: mechanisms and outcomes.

Strategy	Target	Therapeutic outcome	Model used
navarixin (215, 216)	CCR2 and CXCR2	Reduce Ly6Chi monocytes and neutrophils	MI mouse
Celastrol (218)	NLRP3	Anti-inflammatory and fibrotic	MI rat
Calycosin (219)	NLRP3	Anti-inflammatory and fibrotic	MI rat
Quercetin (220)	NETosis	Alleviate reperfusion injury	MI rat
Astragaloside IV (221)	ROS/Caspase-1/GSDMD pathway	Anti-fibrotic	MI mouse
SPMs (132)	Clear apoptotic cells	Anti-inflammatory and anti-fibrotic	MI mouse
Berberine (222)	Wnt5a/β-catenin signal pathway	Promoting Macrophage M2 Polarization	MI mouse
Electroacupuncture (224)	PPARγ/NF-κB signal pathway	Promoting Macrophage M2 Polarization	MI rat
SIS-ECM extracardiac implant (225)	FGF2 and TLR9	Increase VEGF and regulate fibroblast	MI mouse
Curcumin (226)	IL18/TGFβ1/SMAD2/3 signal pathway	Macrophage-Fibroblast Interactions	MI mouse
Tanshinone IIA (227)	HIF-1α	Anti-inflammatory and pro-angiogenic	MI mouse
IL-11 inhibitor (228)	IL11	Reduce myocardial fibrosis	Mouse
MMNP@SB225002 (229)	Neutrophil suppression	Anti-inflammatory and anti-fibrotic	MI mouse
IL-5 nanoparticle (230)	IL-5	Anti-inflammatory and pro-angiogenic	MI mouse
NAM-fused exosomes (231)	ECs	Pro-angiogenic	MI mouse
Chimeric antigen receptor T cells (210)	Tregs	Reduce myocardial fibrosis	HF Mouse
ECs-derived extracellular vesicles (232)	Ly6Chi monocytes and neutrophils	Improve endothelial barrier function	mouse model of IR injury
EVs (233)	miR-146a, miR-21-5p	Inhibit fibroblasts	MI rat
Metformin (236)	AMPK signal pathway	Promoting Macrophage M2 Polarization	Mouse and Human Monocyte
Sialic acid (237)	MCP-1, CCR2, PPARγ	Promoting Macrophage M2 Polarization	MI rat and BMDMs
Dapagliflozin (238)	ROS, MDA and SOD	Inhibit myocardial cell apoptosis	MI mouse
Phosphatidylserine Supplement (239)	PKC-ϵ	Enhance myocardial cell survival	MI mouse
Senolytics (214)	MAO-A	Clear senescent cells	MAO‐A transgenic mice
ECM/Glycopeptide Hybrid Hydrogel (242)	Macrophage -ECs interactions	Promoting Macrophage M2 Polarization	rodent MI model

### 5.1 Temporal precision intervention

This strategy is based on the spatiotemporal dynamics of this network evolution, adopting completely different intervention methods in different phases. The acute phase mainly focuses on inhibiting harmful excessive inflammation while protecting the necessary clearance function. Early use of CCR2 or CXCR2 antagonists in the acute phase can reduce the recruitment of pro-inflammatory Ly6C^hi^ monocytes and neutrophils (215, 216). MSCs also exhibit efficient anti-inflammatory effects, and transplanted MSCs significantly reduce inflammatory macrophages and neutrophils (217). Targeting the NLRP3/IL-1β axis is considered to be able to regulate the early inflammatory level. Studies have used celastrol to inhibit the NLRP3 inflammasome to regulate the early inflammatory storm after MI (218). Calycosin (CA), a flavonoid natural product, can also inhibit the NLRP3 inflammasome, reducing myocardial injury and fibrosis (219). Studies have also suggested that targeted regulation of neutrophils and NETs is an important way to regulate the early inflammatory response after MI. For example, quercetin alleviates reperfusion injury by inhibiting NETs after MI (220). Astragaloside IV reduces the expression of neutrophils by inhibiting the ROS/Caspase-1/GSDMD signaling pathway, improving myocardial fibrosis and cardiac remodeling (221). The repair phase and chronic phase mainly aim to promote inflammation resolution and inhibit pathological fibrosis. Exogenous administration of specialized pro-resolving mediators (SPMs) such as Resolvin D1 and Maresin 1 can actively initiate the inflammation resolution program, enhance apoptotic cell clearance, and promote the conversion of macrophages to the repair phenotype (132). Berberine can also inhibit the Wnt5a/β-catenin pathway in mouse and promote the differentiation of M2 macrophages (222). Electroacupuncture promotes the polarization of macrophages to M2 type and reduces inflammatory injury in myocardial tissue by inhibiting sympathetic nerve remodeling and regulating the PPARγ/NF-κB pathway (223, 224). The development of TGF-β neutralizing antibodies and regulatory drugs targeting fibroblasts and angiogenesis is also a potential therapeutic approach. Epicardial implantation of porcine small intestinal submucosa (SIS)-ECM promotes fibroblast transcription and VEGF production through FGF2 and TLR9 (225). Curcumin regulates the macrophage-fibroblast interaction by inhibiting the IL18/TGFβ1/SMAD2/3 signaling pathway, alleviating myocardial fibrosis (226). Tanshinone IIA upregulates the protein level of hif-1α and vascular endothelial growth factor, inhibits excessive inflammation and increases angiogenesis (227). Recent studies have also found that IL-11 is a more specific pro-fibrotic factor than TGF-β, and monoclonal antibodies targeting IL-11 have shown good prospects in preclinical fibrosis models (228).

### 5.2 Cell-specific targeting

With the emergence of technologies such as engineered cells and nanocarriers, it has become possible to selectively eliminate pathogenic cell subsets or enhance protective cell subsets. Hua designed a nanoparticle loaded with a neutrophil chemotaxis inhibitor (MMNP@SB225002), which can be efficiently delivered to the bone marrow in the early stage of MI, significantly inhibiting neutrophil mobilization and reducing myocardial fibrosis (229). IL-5 nanoparticles promote eosinophil accumulation and angiogenesis, thereby limiting adverse cardiac remodeling after MI (230). Some scholars fused exosomes with neutrophil-derived apoptotic membranes, enhancing the adhesion to inflammatory ECs, replicating the recruitment mechanism of neutrophils at the myocardial injury site, and also promoting angiogenesis in MI mice (231). Fibroblast activation protein (FAP) is a relatively specific marker of activated myofibroblasts. The use of FAP-targeted CAR-T cells and FAP antibody-drug conjugates has successfully reduced cardiac fibrosis in preclinical studies (210). Nasal administration of ECs-derived extracellular vesicles (EVs) combined with hydrogel reduces the expression of Ly6C^hi^ monocyte/macrophages and neutrophils, inhibits inflammatory responses, improves endothelial barrier function, and increases microvascular density in the injured area (232). EVs derived from M2 macrophages are rich in anti-inflammatory miRNAs, which can be taken up by cardiac fibroblasts to inhibit their activation (233).

### 5.3 Regulating cell metabolism and fate

Cell phenotype and function are closely coupled with their metabolic state. The function of key cells can be altered by pharmacological reprogramming of their metabolic pathways. Pro-inflammatory M1 macrophages rely on glycolysis (234), while reparative M2 macrophages are more dependent on oxidative phosphorylation and fatty acid oxidation (235). The use of metformin or AMPK activators can promote oxidative metabolism and induce the conversion of macrophages to M2 phenotype (236). Sialic acid (SA) reduces the expression of MCP-1 and CCR2, decreases the levels of inflammatory factors TNF-α, IL-1α and IL-1β, and activates PPARγ to promote M2 polarization of macrophages (237). Dapagliflozin (DAPA) can reduce oxidative stress to protect cardiomyocytes, characterized by decreased levels of ROS and malondialdehyde (MDA), while restoring superoxide dismutase (SOD) activity, thereby inhibiting cardiomyocyte apoptosis (238). Supplementation with phosphatidylserine can significantly upregulate protein kinase Cϵ (PKC-ϵ) and improve the survival rate of cardiomyocytes (239). Sustained activation of some pro-fibrotic signaling pathways can lead to senescence of myofibroblasts. Selective clearance of these senescent cells using senolytics (such as dasatinib combined with quercetin) can reduce fibrosis and improve cardiac function (214). Palmitoylation, a key lipid post-translational modification, regulates macrophage signal transduction, inflammatory activation, and interactions with stromal cells (240). Studies have shown that knockout of the palmitoylation gene ZDHHC18 reduces epithelial–mesenchymal transition and alleviates fibrotic progression (241). ECM/glycopeptide hybrid hydrogels construct niches to reproduce the structure of native ECM, attract host cell homing, control macrophage differentiation, and promote ECs proliferation by enhancing macrophage-ECs interactions (242).

## 6 Conclusion and outlook

This review systematically outlines the core framework of the “immunocyte-mesenchymal cell bidirectional regulatory network,” transcending the traditional perception of repair as a simple linear cascade. Research clearly reveals that immune cells such as neutrophils, monocytes/macrophages, and lymphocytes, along with stromal cells like cardiac fibroblasts and ECs, form a highly dynamic spatiotemporal ecosystem through a complex interplay of soluble factors, stromal signals, and direct contact signals. The initial equilibrium of this network underpins effective repair and stable scar formation. Its sustained imbalance—manifesting as impaired inflammation resolution, amplified repair signals, and mechanical positive feedback loops—constitutes the fundamental pathomechanism driving progressive ventricular dilatation and diffuse fibrosis, ultimately leading to heart failure. This insight also provides a roadmap for future precision therapies. Traditional broad-spectrum anti-inflammatory or unidirectional pathway inhibition strategies have limited efficacy due to their inability to distinguish between injurious and protective inflammation, coupled with a lack of spatiotemporal specificity. Future breakthrough therapies will inevitably be network-oriented and precision-based. On one hand, cutting-edge technologies will systematically decode the cellular ecosystem of the human heart post-MI to identify key therapeutic targets. On the other hand, developing intelligent delivery systems such as nanoparticles and engineered exosomes will enable precise drug delivery to specific cells at specific times. Interdisciplinary collaboration will drive progress in this field, ultimately achieving precision medicine based on specific network characteristics to reverse the progression of heart failure.
